# Low apoA-I is associated with insulin resistance in patients with impaired glucose tolerance: a cross-sectional study

**DOI:** 10.1186/s12944-017-0446-1

**Published:** 2017-04-04

**Authors:** Xiaomeng Feng, Xia Gao, Zhi Yao, Yuan Xu

**Affiliations:** Department of Endocrinology, Beijing Chao-Yang Hospital, Capital Medical University, Beijing, 100020 People’s Republic of China

**Keywords:** Insulin resistance, Apolipoprotein A-I, Impaired glucose tolerance

## Abstract

**Background:**

Low apolipoprotein A-I (apoA-I) is an independent risk factor for atherosclerotic cardiovascular diseases. Insulin resistance predicts the progression of abnormal glucose metabolism, which is the main cause of atherosclerotic cardiovascular disease. In this study, we assessed the potential association between apoA-I levels and insulin resistance in patients with impaired glucose tolerance (IGT) and the possible link between apoA-I and IGT.

**Methods:**

This study evaluated a cross-sectional study of 108 participants with impaired glucose tolerance (IGT group) and 84 controls (control group). ApoA-I and clinical characteristics were measured, and a homeostasis model assessment of insulin resistance (HOMA-IR) was calculated.

**Results:**

The IGT group exhibited significantly lower apoA-I and higher HOMA-IR levels than the control group (apoA-I: 1.37 ± 0.36 vs 1.57 ± 0.39 g/L; HOMA-IR: 4.21 ± 1.56 vs 2.15 ± 0.99; *P* < 0.001 for both). ApoA-I was negatively correlated with HOMA-IR in both the IGT and control groups (IGT group: *r* = −0.269, *P* = 0.005; control group: *r* = −0.262, *P* = 0.016). Multiple stepwise regression analysis showed that low apoA-I levels (*β* = −1.470, *P* = 0.002) were independently correlated with high HOMA-IR levels in the IGT group. Moreover, logistic regression analysis identified that low apoA-I was an independent influencing factor for IGT (*β* = −1.170, OR = 0.310, *P* = 0.007).

**Conclusions:**

ApoA-I is inversely associated with insulin resistance in patients with impaired glucose tolerance, and low apoA-I is an independent risk factor for impaired glucose tolerance. These results indicate that apoA-I plays an important role in regulating insulin sensitivity and glucose metabolism in patients with IGT.

## Background

Insulin resistance predicts the development of type 2 diabetes [1] and atherosclerotic cardiovascular disease (ASCVD) [2] and begins to increase considerably before the onset of diabetes. Impaired glucose tolerance (IGT), which is characterized by high postprandial blood glucose levels, causes the development of type 2 diabetes [3] and ASCVD [4]. Postprandial blood glucose levels are frequently associated with elevated insulin resistance, which may have a more damaging effect on the vasculature including the activation of inflammatory pathways, increased oxidative stress, an extensive procoagulant state, and abnormal vasomotion [5].

High-density lipoprotein cholesterol (HDL-C) is involved in cardiovascular protection [6]. Apolipoprotein A-I (apoA-I) is the major apolipoprotein constituent of HDL-C [6]. Both HDL-C and apoA-I play important roles in reverse cholesterol transport and exhibit anti-atherogenic activities, including anti-thrombotic, anti-oxidative, and anti-inflammatory activities, which are independent of their reverse cholesterol transport activities [7]. Based on the results of many epidemiological studies, HDL-C has been considered an independent negative risk factor for ASCVD; however, in the INTERHEART STUDY, apoA-I levels were shown to have a stronger effect in preventing the development of ASCVD than HDL-C levels [8]. This finding suggests that it is necessary to focus not simply on decreases in HDL-C levels but on the fact that decreases in apoA-I levels are important for the induction of ASCVD. Moreover, apoA-I has been documented to independently promote insulin secretion and glucose uptake [9] and to be negatively correlated with insulin resistance in patients with type 2 diabetes [10].

Studies have shown that insulin secretion and islet β-cell function are elevated several years before the onset of diabetes and then decrease until the time of diagnosis [11]. A compensatory period (i.e., a compensatory increase in insulin production that is secondary to high insulin resistance and the elevated secreting load of islet β-cells with subtle changes in glucose levels) has been identified before the onset of diabetes, and insulin production decreases after the diagnosis of diabetes [11]. Insulin resistance might be considered to be responsible for the increase in the secreting load of islet β-cells and insulin secretion during the compensatory period. Furthermore, reduced levels of apoA-I occur years before the development of type 2 diabetes [12]. Therefore, it is of interest to assess the possible association between apoA-I and insulin resistance in patients with impaired glucose tolerance and the potential correlation between apoA-I and IGT. However, these associations have not been well-characterized. In this study, we assessed the possible association between apoA-I and insulin resistance in patients with impaired glucose tolerance and the link between apoA-I and the probability of being IGT.

## Methods

### Subjects

All participants (both genders, ranging in age from 30 to 70 years) were recruited from March 2015 to March 2016.

In total, 108 patients with impaired glucose tolerance were recruited for this study from a group of outpatients at the Department of Endocrinology, Beijing Chao-Yang Hospital, Capital Medical University, Beijing, China. Participants diagnosed with IGT as defined by the American Diabetes Association criteria [13] were eligible for the study. The following exclusion criteria for the IGT group were applied: normal glucose tolerance, impaired fasting glucose, and diabetes.

Eighty-four people were recruited to the control group from the community and people undergoing routine medical check-ups. None of these people were diagnosed with prediabetes (including impaired glucose tolerance and impaired fasting glucose) or diabetes.

Moreover, people with hypertension, coronary artery disease, endocrine disease, systemic inflammatory disease, infectious disease, cancer, liver or renal function impairment, pregnancy or lactation were excluded from both groups. People taking agents known to influence glucose or insulin metabolism and/or people being treated with lipid-lowering drugs were also excluded from both groups.

The study protocol was approved by the Medicine and Pharmacy Ethics Committee of Beijing Chao-Yang Hospital, Capital Medical University. Written informed consent was obtained from each participant.

### Clinical and biochemical measurements

A standard questionnaire was used to collect information about the participants’ health status and medications. Height and weight were measured without shoes and in light clothing to the nearest 0.1 cm and 0.1 kg, respectively, by the same trained group. Body mass index (BMI) was calculated as weight (kg) / [height (m)]^2^. Blood pressure was measured using a calibrated standard mercury sphygmomanometer. All readings were measured from the non-dominant arm after a 5-min resting period with the patients in the sitting position.

Fasting blood samples were collected in the morning after an 8-h overnight fast. All patients underwent 75 g oral glucose tolerance tests (OGTT). Blood samples were collected at 0 min and 120 min following the OGTT. Total cholesterol (TC), HDL-C, low-density lipoprotein cholesterol (LDL-C), triglycerides (TG), apoA-I, apolipoprotein B (apoB), fasting blood glucose (FBG), 2-h postchallenge glucose (2hPG), glycated hemoglobin (HbA1c) and fasting insulin (FINS) levels were measured in the Central Laboratory of Beijing Chao-Yang Hospital, Capital Medical University.

Serum insulin levels were determined by the electrochemiluminescence method using an Elecsys-2010 Automatic Electrochemical Immuno-analyser (Roche Corporation). Blood glucose levels were measured using the hexokinase-UV/NAD method (Olympus). Blood lipids were measured as follows: TC levels were measured using the cholesterol oxidase-HDAOS method (Wako); TG levels were measured using the GPO-HDAOS glycerol blanking method (Wako); HDL-C levels were measured using the immunoinhibition (direct) method (Wako); LDL-C levels were measured using the selective protection enzymatic (direct) method (Wako), and apoA-I and apoB levels were measured using the immunoturbidimetric method (Olympus). Serum glucose and lipid levels were analysed using an Olympus AU5400 Automatic Biochemistry Analyser (Olympus Corp, Japan). HbA1c analysis was performed by high-performance liquid chromatography using a HLC-723 G8 Automatic Analyser (Tosoh Corp, Japan). Insulin resistance was measured using the following method: homeostasis model assessment of insulin resistance (HOMA-IR) = FINS (mIU/L) * FBG (mmol/L) / 22.5.

### Statistical analysis

All analyses were performed using Statistical Package for Social Sciences version 19.0 (SPSS, Inc., Chicago, IL, USA). The normality of the data distribution was verified using the Kolmogorov-Smirnov test. Normally distributed data were expressed as the means ± standard deviations. Non-normally distributed data were expressed as medians with 25th and 75th percentiles. Comparisons of the clinical and biochemical markers between the two groups were performed using the independent sample *t* test and the Mann-Whitney *U* Test. Proportions were analysed using the chi-squared test. Association was tested using Pearson’s correlation coefficient analyses, multiple stepwise regression analyses, and logistic regression analysis. In all statistical tests, *P* values < 0.05 were considered significant, and all tests were two-sided.

## Results

### Clinical characteristics of individuals in the IGT and control groups

The clinical characteristics of the participants are summarized in Table 1. The participants in both groups were similar in sex, age, systolic blood pressure (SBP), diastolic blood pressure (DBP), TC, and apoA-I/HDL-C (*P* > 0.05 for all). The levels of BMI (*P* < 0.001), LDL-C (*P* = 0.024), TG (*P* < 0.001), apoB (*P* = 0.030), FBG (*P* < 0.001), 2hPG (*P* < 0.001), HbA1c (*P* < 0.001) and FINS (*P* < 0.001) were higher and the levels of HDL-C (*P* < 0.001) were lower in the IGT group than in the control group.

**Table 1:** Clinical characteristics of the study participants

Parameters	IGT group (*n* = 108)	Control group (*n* = 84)	*P* value
Sex (M/F)	65/43	44/40	0.279
Age (years)	57.31 ± 7.21	56.83 ± 7.55	0.651
BMI (kg/m^2^)	26.43 ± 4.83	24.26 ± 3.44	0.001
SBP (mmHg)	128.11 ± 6.86	126.46 ± 6.89	0.101
DBP (mmHg)	75.62 ± 6.50	74.14 ± 7.38	0.142
TC (mmol/L)	5.15 ± 1.06	4.92 ± 0.91	0.109
HDL-C (mmol/L)	1.34 ± 0.27	1.58 ± 0.37	<0.001
LDL-C (mmol/L)	3.10 ± 0.74	2.85 ± 0.75	0.024
TG (mmol/L)	2.37 (1.99, 2.79)	1.09 (0.71, 1.48)	<0.001
ApoA-I (g/L)	1.37 ± 0.36	1.57 ± 0.39	<0.001
ApoB (g/L)	0.97 ± 0.22	0.90 ± 0.23	0.030
ApoA-I/HDL-C	0.99 (0.91, 1.10)	0.99 (0.85, 1.11)	0.821
FBG (mmol/L)	6.30 (5.60, 6.98)	5.58 (5.24, 5.82)	<0.001
2hPG (mmol/L)	9.30 (8.63, 9.80)	6.50 (6.08, 6.73)	<0.001
HbA1c (%)	6.30 (6.00, 6.40)	5.90 (5.60, 6.10)	<0.001
FINS (mIU/L)	15.70 (10.80, 18.30)	8.17 (5.39, 11.97)	<0.001
HOMA-IR	4.21 ± 1.56	2.15 ± 0.99	<0.001

IGT group, patientswith impaired glucose tolerance; Control group, control subjects; *BMI* body mass index, *SBP* systolic blood pressure, *DBP* diastolic blood pressure, *TC* total cholesterol, *HDL-C* high-density lipoprotein cholesterol, *LDL-C* low-density lipoprotein cholesterol, *TG* triglycerides, *apoA-I* apolipoprotein A-I, *apoB* apolipoprotein B, *FBG* fasting blood glucose, *2hPG* 2-h postchallenge glucose, *HbA1c* glycated hemoglobin, *FINS* fasting insulin, *HOMA-IR* homeostasis model assessment of insulin resistance

### Levels of apoA-I in the IGT and control groups

**Fig. 1:**
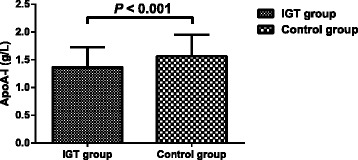
Apolipoprotein A-I (apoA-I) levels measured in the study participants. The values are expressed as the means ± standard deviations. IGT group, patients with impaired glucose tolerance (*n* = 108); Control group, control subjects (*n* = 84)

The levels of apoA-I were significantly lower in the IGT group than those in the control group (1.37 ± 0.36 vs 1.57 ± 0.39 g/L, *P* < 0.001) (Fig. 1).

### Values of HOMA-IR in the IGT and control groups

**Fig. 2:**
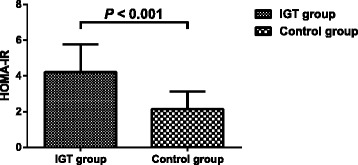
Homeostasis model assessment of insulin resistance (HOMA-IR) of the study participants. The values are expressed as the means ± standard deviations. IGT group, patients with impaired glucose tolerance (*n* = 108); Control group, control subjects (*n* = 84)

The values of HOMA-IR were significantly higher in the IGT group than those in the control group (4.21 ± 1.56 vs 2.15 ± 0.99, *P* < 0.001) (Fig. 2).

### Correlation between apoA-I and HOMA-IR

**Fig. 3:**
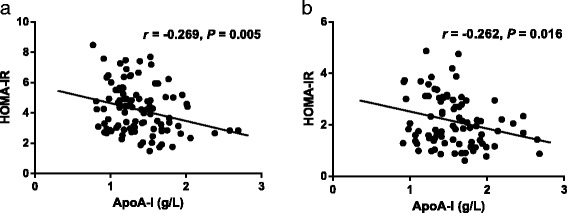
Correlations between apoA-I levels and HOMR-IR in the IGT group (**a**) and in the control group (**b**). ApoA-I, apolipoprotein A-I; HOMR-IR, homeostasis model assessment of insulin resistance; IGT group, patients with impaired glucose tolerance (*n* = 108); Control group, control subjects (*n* = 84)

ApoA-I was negatively correlated with HOMA-IR in both the IGT and control groups (IGT group: *r* = −0.269, 95% confidence interval: −0.419 to −0.105, *P* = 0.005, (Fig. 3a); control group: *r* = −0.262, 95% confidence interval: −0.426 to −0.082, *P* = 0.016; Fig. 3b).

### Multiple regression analyses of anthropometric parameters and lipid profile associated with HOMA-IR

Multiple stepwise regression analyses were performed to determine which parameters were independently associated with HOMA-IR in the two groups. The results showed that after adjusting for anthropometric parameters and lipid profile, the increased TG levels (*P* = 0.002) and the decreased apoA-I levels (*P* = 0.002) were independently related to high HOMA-IR in the IGT group (Table 2) and the elevated BMI levels (*P* = 0.010) were independently associated with high HOMA-IR in the control group (Table 3). The model in the IGT group had an adjusted R-squared value of 0.174 (F = 9.855 and *P* < 0.001), and the model in the control group had an adjusted R-squared value of 0.069 (*F* = 7.039 and *P* = 0.010).

**Table 2:** Multiple regression of parameters associated with HOMA-IR in patients with IGT (*n* = 108)

Parameters	*β*	SE	Standardized *β*	95% CI	*P* value
Constant	4.788	0.752		3.282 ~ 6.274	<0.001
TG (mmol/L)	0.540	0.166	0.323	0.209 ~ 0.870	0.002
ApoA-I (g/L)	−1.470	0.456	−0.319	−2.382 ~ −0.558	0.002

*HOMA-IR* homeostasis model assessment of insulin resistance, *IGT* impaired glucose tolerance; *SE* standard error, *CI* confidence interval, *TG* triglycerides, *apoA-I* apolipoprotein A-I. Adjustment for age, sex, body mass index (BMI), systolic blood pressure (SBP), diastolic blood pressure (DBP), total cholesterol (TC), high-density lipoprotein cholesterol (HDL-C), low-density lipoprotein cholesterol (LDL-C), TG, apoA-I, and apolipoprotein B (apoB)

**Table 3:** Multiple regression of parameters associated with HOMA-IR in controls (*n* = 84)

Parameters	*β*	SE	Standardized *β*	95% CI	*P* value
Constant	0.167	0.754		−1.332 ~ 1.667	0.825
BMI (kg/m^2^)	0.082	0.031	0.283	0.020 ~ 0.143	0.010

*HOMA-IR* homeostasis model assessment of insulin resistance, *SE* standard error, *CI* confidence interval, *BMI* body mass index. Adjustment for age, sex, BMI, systolic blood pressure (SBP), diastolic blood pressure (DBP), total cholesterol (TC), high-density lipoprotein cholesterol (HDL-C), low-density lipoprotein cholesterol (LDL-C), triglycerides (TG), apolipoprotein A-I (apoA-I), and apolipoprotein B (apoB)

### Logistic regression analysis of the anthropometric parameters and lipid profile associated with IGT

The adjusted results of a logistic regression analysis of the associations between the anthropometric parameters and the lipid profile and IGT are shown in Table 4. ApoA-I and BMI were independently associated with IGT: apoA-I (*P* = 0.007) was inversely associated and BMI (*P* = 0.008) was positively associated with IGT.

**Table 4:** Logistic regression of anthropometric parameters and lipid profile associated with IGT (*n* = 182)

	*β*	SE	OR	95% CI	*P* value
Constant	−0.784	1.296	0.457		0.546
ApoA-I (g/L)	−1.170	0.433	0.310	0.133 ~ 0.725	0.007
BMI (kg/m^2^)	0.109	0.041	1.115	1.029 ~ 1.209	0.008

*IGT* impaired glucose tolerance, *SE* standard error, *OR* odds ratio, *CI* confidence interval, *apoA-I* apolipoprotein A-I, *BMI* body mass index. Adjustment for age, sex, BMI, systolic blood pressure (SBP), low-density lipoprotein cholesterol (LDL-C), and apoA-I

## Discussion

In the present study, patients with impaired glucose tolerance presented significantly lower levels of HDL-C and apoA-I than the controls. HDL-C is an anti-atherogenic lipoprotein. ApoA-I is a major functional component of HDL-C and is extensively involved in the cardiovascular protective effects of HDL-C [6]. HDL-C and apoA-I transport cholesterol from cells into peripheral tissues, reduce oxidative stress, suppress inflammatory pathways [7], neutralize procoagulant properties of anionic phospholipids and attenuate the excessive stimulation of blood coagulation [14], and prevent LDL-C oxidation by removing oxidized phospholipids from LDL-C and from arterial wall cells [15]. Therefore, the administration of apoA-I has been proposed for use as a potential therapeutic strategy to protect the cardiovascular system [16, 17]. Transgenic mice over-expressing apoA-I exhibit high HDL-C levels and low vascular lesions [18]. Laboratory studies have shown that apoA-I reduces the lipid and macrophage content of arteries and decreases lesion formation in mouse and rabbit models of atherosclerosis [19, 20]. ApoA-I infusion has been documented to reduce atheroma volume in patients with coronary atherosclerosis compared with baseline [21]. In addition, pharmacological strategies that target apoA-I, including the upregulation of its production with the bromodomain and extraterminal protein inhibitor RVX-208, the development of short peptide sequences that mimic its action, and its administration as a component of reconstituted HDL-C (containing apoA-I as its only protein and a phospholipid as its only lipid) have beneficial effects on inflammatory factors that are known to be involved in atherosclerosis and plaque stability [16, 22]. However, it has been demonstrated that TaqIB polymorphism in the cholesterol ester transfer protein gene has a significant impact on HDL-C levels, while the effect of a 75G/A polymorphism in the apoA-I gene was not significant [23]. Although several clinical studies have indicated that low apoA-I levels are an independent risk factor for ASCVD [24–26], the underlying mechanism linking apoA-I with the delay of atherosclerotic plaque progression remains unknown. The results of various studies have supported the notion that apoA-I protection against cardiovascular events might at least in part be mediated through improving insulin sensitivity.

In our study, the IGT group exhibited higher HOMA-IR and FINS than the control group, a finding that might support the notion that IGT promotes the progression of ASCVD due to insulin resistance and hyperinsulinemia. Hyperinsulinemia caused by insulin resistance directly stimulates the in vitro migration of neutrophils and monocytes in response to chemokines that are secreted by atherosclerotic plaques [27]. Hyperinsulinemia might promote atherosclerotic plaque necrosis by accelerating macrophage death [28]. In particular, hyperinsulinemia induces the production of matrix metalloproteinase-9 (MMP-9), which provokes plaque instability and rupture [29]. Pharmacological and behavioural treatments to reduce insulin resistance have been shown to repress MMP-9 secretion [30]. Moreover, insulin resistance might intensify a serious atherothrombotic state by elevating platelet resistance to antiaggregating agents [31] and accelerating the production of procoagulatory factors [30]. Thus, the evidence strongly supports an emerging role of insulin resistance in plaque instability and rupture.

The present cross-sectional study identified the inverse association between apoA-I and insulin resistance in patients with impaired glucose tolerance; this finding is similar to that of a previous study in patients with type 2 diabetes [10]. Recent studies have reported that HDL-C and apoA-I improve pancreatic β-cell function. In human islet cell culture and animal studies, exogenous HDL-C has been found to attenuate the inflammation-induced apoptosis of pancreatic β-cells [9]. The infusion of exogenous reconstituted HDL-C potentiated both insulin secretion and skeletal muscle glucose uptake in a small trial with type 2 diabetic patients [32]. ApoA-I treatment was found to increase glucose-stimulated insulin secretion in mice that were fed a high-fat diet [33, 34] and ameliorated β-cell dysfunction by inhibiting β-cell apoptosis [9, 35]. The favourable effects of apoA-I on improving insulin sensitivity have been further investigated in human skeletal muscle cells and adipocytes, in which both HDL-C and apoA-I promoted glucose uptake independently of insulin stimulation [32, 36, 37]. The mechanism connecting low apoA-I to high insulin resistance has been unclear. However, recent studies have supported the hypothesis that apoA-I might exert beneficial effects on ameliorating insulin resistance through different pathways. Recently, a new concept has been accepted suggesting that insulin resistance might primarily start in the vascular endothelium [38]. Endothelial nitric oxide synthase (eNOS) dysfunction might reduce microcirculatory blood flow and decrease the delivery of insulin within hormone-sensitive organs. Insulin-mediated glucose uptake has been found to be inhibited in eNOS^−/−^ mice compared with wild type mice [39]. Thus, the restoration of eNOS function can decrease insulin resistance [40]. It has been proposed that apoA-I is fundamental for the process by which HDL-C activates eNOS [41]. ApoA-I binding to the scavenger receptor-BI is required for the HDL-C activation of eNOS [41]. Furthermore, apoA-I is responsible for the effects of endothelial cell migration. ApoA-I has been proven to prevent endothelial apoptosis that is induced by oxidized-LDL-C [42] and tumour necrosis factor α [43]. It has been suggested that inflammation might be crucial for the development of insulin resistance and β-cell dysfunction [44]. Inflammatory cytokines and acute-phase reactants are positively correlated with insulin resistance in patients with metabolic syndrome [45]. The anti-inflammatory effects of apoA-I have been observed in both in vitro and in vivo studies. Reconstituted HDL-C has been found to inhibit the expression of vascular cell adhesion molecule-1, intercellular adhesion molecule-1, and E-selection in activated endothelial cells growing in tissue culture [46]. In macrophages, apoA-I has been shown to prevent inflammation by suppressing cluster of differentiation 40 L-induced activation of nuclear factor-κ-gene binding [47]. A recent study has reported that changes in apoA-I but not in HDL-C are negatively correlated with changes in high-sensitivity C-reactive protein (hs-CRP), which is a predictor of the development of cardiovascular events [48]. The increase of apoA-I that follows pitavastatin treatment is an independent predictor of decreased levels of hs-CRP [49]. Hence, apoA-I might decrease insulin resistance by improving vascular endothelium function and inhibiting inflammation.

Therefore, we speculated that decreased insulin resistance due to increased apoA-I levels may partially explain the protective effects of apoA-I on the cardiovascular system that have been observed in clinical trials [24–26]. Increased insulin sensitivity that is mediated through high apoA-I levels favours the transport of glucose from the circulation into tissues and has potential clinical relevance in terms of reducing cardiovascular complications by removing excess glucose from the circulation and in providing adequate glucose to tissues for energy production, particularly in the context of cardiovascular events [50].

Importantly, the finding that apoA-I is negatively related to insulin resistance might partially explain our result that low apoA-I is an independent factor influencing impaired glucose tolerance. Recent studies have documented that insulin resistance is a major determinant of the development and progression of abnormal glucose metabolism [1]. Increased insulin resistance drives a compensatory increase in insulin secretion during the early stage of abnormal glucose metabolism [11]. However, the chronic overload of islet β-cells will lead to the deterioration of β-cell function, if sustained. When insulin secretion is insufficient and blood glucose levels rise, IGT and diabetes ultimately become overt. Therefore, IGT might be the result of chronic exposure to severe insulin resistance, which is at least partly induced by low apoA-I levels and which portends the earlier initiation of hyperglycaemia [10]. Therefore, low apoA-I levels might be an independent risk factor for IGT.

The limitations of our study are as follows. First, our study population was limited to Chinese individuals. Therefore, our findings may not be directly applicable to other populations. Second, our sample size was small; thus, our findings were not powerful enough to account for potentially confounding factors in our analyses, and our results might have been improperly influenced by some outliers due to the small sample size. Third, the cross-sectional design of the present study does not allow us to determine the existence of a causal relationship but rather provides evidence for the association between low apoA-I levels and high insulin resistance in patients with impaired glucose tolerance. Our study certainly raises credible hypotheses that remain be confirmed and extended by future prospective cohort and mechanistic studies. Fourth, our study estimated insulin resistance based on HOMA-IR rather than by precise methods, such as the hyperinsulinemic euglycaemic clamp technique.

## Conclusions

Here, we report a negative association between apoA-I and insulin resistance in patients with impaired glucose tolerance and demonstrate a correlation between low apoA-I levels and impaired glucose tolerance. These results indicate that apoA-I plays an important role in regulating insulin sensitivity and glucose metabolism in patients with impaired glucose tolerance.

## Abbreviations

2hPG: 2-hour postchallenge glucose
ApoA-I: Apolipoprotein A-I
apoB: Apolipoprotein B
ASCVD: Atherosclerotic cardiovascular disease
BMI: Body mass index
DBP: Diastolic blood pressure
eNOS: Endothelial nitric oxide synthase
FBG: Fasting blood glucose
FINS: Fasting insulin
HbA1c: Glycated hemoglobin
HDL-C: High-density lipoprotein cholesterol
HOMA-IR: Homeostasis model assessment of insulin resistance
hs-CRP: High-sensitivity C-reactive protein
IGT: Impaired glucose tolerance
LDL-C: Low-density lipoprotein cholesterol
MMP-9: Matrix metalloproteinase-9
OGTT: Oral glucose tolerance test
SBP: Systolic blood pressure
TC: Total cholesterol
TG: Triglycerides
